# Transactional Sex and HIV Risk among Adolescent School Girls in Ethiopia: Mixed Method Study

**DOI:** 10.1155/2019/4523475

**Published:** 2019-06-27

**Authors:** Liyuwork Mitiku Dana, Yohannes Mehretie Adinew, Mitike Molla Sisay

**Affiliations:** ^1^College of Health Sciences and Medicine, Wolaita Sodo University, Sodo, Ethiopia; ^2^School of Public Health, College of Health Science, Addis Ababa University, Addis Ababa, Ethiopia

## Abstract

**Background:**

Young people in Sub-Saharan Africa are affected by HIV pandemic to a greater extent than elsewhere. Transactional sex among adolescent school girls with older men commonly called “sugar daddies” is one of the major factors fueling the spread of the infection due to the extended sexual network. Thus, this study aimed to assess the magnitude and factors associated with transactional sex among adolescent girls and “sugar daddies” in relation to HIV/AIDS.

**Methods:**

Mixed method cross-sectional study was done among 620 female students in Hawassa town, South Ethiopia, from September 2010 to May 2011. A structured questionnaire and in-depth interview check list were used to collect the quantitative and qualitative data, respectively. Survey participants were selected randomly from five preparatory schools whereas ten in-depth interview participants were recruited by a snowball sampling technique from the same schools. Data were entered using Epi-Info and analyzed by SPSS. A descriptive statistics followed by multivariable logistic regression analyses were conducted to identify factors associated with transactional sex with “sugar daddy”. Both crude and adjusted odds ratios with 95% confidence interval were reported. We used OpenCode software for coding and categorizing the in-depth interviews and quotes that represent the informants opinion were used to support the quantitative findings.

**Results:**

A substantial number of female students, 71(11.5%), reported to have had transactional sex with older men. Most of the respondents who dated “sugar daddies” (93%) had multiple sexual partners concurrently and sequentially, and among them, only 22.7% had consistent condom use. Girls who were in older age group [OR (CI) 6.87 (3.48-13.58)], who had lost both parents [OR (CI) 2.99 (1.14-7.84)], had perceived less economic status [OR: 25.41; 95% CI: 7.80-82.76] and engaged in substance abuse [OR (CO) 5.8 (2.1-15.77)] had higher odds of practicing transactional sex with “sugar daddies”. In-depth interviewed participants also revealed that they were involved in transactional sex for monetary while having concurrent and subsequent sexual network with their schoolmates and other young partners.

**Conclusion:**

Transactional sex among female students was high, and the sexual network they had with the young men put young people in the network at risk of HIV and other sexually transmitted infections. Therefore, HIV prevention programs shall focus on transactional sex among adolescent school girls to halt transmission of HIV among the generation.

## 1. Background

According to World Health Organization (WHO) Acquired Immune Deficiency Syndrome (AIDS) epidemic updated report of 2015, an estimated of 36.7 million people were infected by HIV worldwide. Young people in Sub-Saharan Africa (SSA) are affected by the pandemic to a greater extent than young people elsewhere [1]. There were 2.1 million new infections worldwide with 1.1 million (52%) occurring in SSA. In the same year, 1.1 million people died of AIDS-related illnesses globally, nearly three-quarters were (1.4 million) in SSA [2]. Ethiopia is one of highly hit nations; in 2014 an estimated of 1.14 million people were living with the virus [3].

HIV infection and AIDS mortality affect primarily the young, most productive segment of the population. Among newly infected people in SSA in 2016, 40% belongs to the age group of 15–24 years and the majority (60%) of these infections occurred among young girls [4]. The chance of getting HIV/AIDS among young females is increasing rapidly over time than males of the same age group [5]. In Ethiopia, HIV/AIDS prevalence is higher in young females than males [3].

One of the commonest sexual relationship among young girls especially those from lower economic echelon is sexual relation with older men from a better economic status who are commonly known as “sugar daddies” [6–8]. Transactional sex (TS) or sexual relation with “sugar daddies” is defined as heterosexual intercourse with a nonmarital partner ten or more years older [6, 9, 10], primarily characterized by relationships between younger women and older men [7, 11]. Since older men might have more sexual experience than the young women, their odd of getting infected is high [12]. This primarily relates to unsafe sex and multiple sexual partners [3, 5, 13–15]. The extent of practicing in sexual relation between young females and “sugar daddies” is more prevalent in urban than rural areas [16, 17]. The higher risk of HIV infection among young girls can go beyond them due to sexual networking that makes the whole generation vulnerable [18].

TS related sexual behaviors are not well explored in Ethiopia. Thus, this study sought to increase understanding of the dynamics of sexual relationship of adolescent girls with older men and its consequent risky sexual behaviors and to understand their decision-making in relation to HIV/AIDS.

## 2. Methods

### 2.1. Study Design and Setting

Mixed cross-sectional study, survey followed by in-depth interviews, was done in Hawassa town from September 2010 to May 2011. Hawassa is located 275 Km to south of Addis Ababa, the capital city of Ethiopia. According to 2007 census, the total population of the town was 259,803 and of them, 125,685 were females. The total number of adolescents and youth in the age group of 15-24 was estimated to be around 50,063 [19]. The town is one of the most popular resort areas in Ethiopia visited by many foreigners and national tourists.

### 2.2. Sample Size and Sampling Procedures

Study populations of the quantitative data were preparatory students (grade 11-12) from five randomly selected schools in Hawassa town in 2010/11 academic year. The sample size was calculated using single population proportion formula with assumptions of 95% level of confidence, 5% margin of error, and 25% proportion of TS with “sugar daddies” [20]. Design effect of 2 and an addition of 10% for possible nonresponse constituted a total sample size of 634. Study sample was proportionally selected by computer generated random sampling method from list of students obtained from each of the five randomly selected schools. Selected students were contacted through their respective schools and were oriented about the study and their random selection. Appointment was made for day of data collection after deep discussion that removed their doubts and cleared their misunderstandings. In-depth interview participants who revealed that they engaged in TS with “sugar daddies” were recruited from the same schools using nonprobabilistic snowball sampling technique.

### 2.3. Data Collection Tools and Procedures

A structured self-administered questionnaire was used to collect quantitative data. The instrument was adopted from previous studies [14, 21–27]. It was designed in English and translated to local language, Amharic, and then back to English by third person to check for internal consistency. Amharic version of the questionnaire was used for data collection. Ten female diploma nurses facilitated data collection under two experienced supervisors. Data collection in each school took place within a day to avoid possible information contamination. A total of 10 in-depth interviews were conducted among students who involved in transactional sex with “sugar daddies”. In-depth interview was held until information saturation was reached and average time per interview was 60 minutes. A semistructured open ended topic guide was used to facilitate the interview. Themes in the topic guide were mainly related to TS with “sugar daddies” in relation to HIV, sexual networking, and other risky sexual behaviors. In-depth interviews were tape recorded after obtaning informed consent from each interviewee.

### 2.4. Data Quality Management

Two days intensive training was given to data collectors and supervisors. Moreover, the instrument was pretested on 32 preparatory students from the non-participated school and necessary amendments were made prior to actual data collection. In-depth interviews were took place other than school hours in quiet places to ensure privacy and confidentiality. The interviews were conducted by the same-gender composition, female interviewer, because this makes sharing ideas easier, especially on highly sensitive topics like the current study topic.

### 2.5. Data Processing and Analysis

Questionnaires were checked for completeness and entered into Epi-Info and then exported to SPSS for further analysis. Descriptive statistics were first done. Bivariate and multivariate logistic regression models were then carried out to identify factors associated with transactional sex with "sugar daddy'. Odds ratios and corresponding 95% confidence intervals were reported and variables with p-value < 0.05 were considered significant factors. Tape rRecords and notes from in-depth interviews were transcribed and translated verbatim from local language to English by individuals fluent in both languages after reviewing and familiarization. Translated data were saved in a text format and analyzed by OpenCode software. Coding process involved identifying major themes in each of the transcripts. Identified themes were compared across transcripts to determine differences and similarities in perspectives of study participants on TS and driving factors influencing their decisions. Eight different categories were developed from which one general theme was constituted (Table 1).

### 2.6. Operational Definition and Measurement

- Transactional sex: heterosexual intercourse in exchange for money and/or material goods.
- “Sugar daddy”: older men having sexual relationship with young girls in exchange for money and/or material goods. Students who ever had such relationship with a partner that had at least 20 years of age gap were considered to have sexual relationship with “sugar daddies”.
- Sexual violence: any sexual act, attempt to obtain a sexual act, unwanted sexual comments, or advances directed against a person's sexuality using coercion, by any person regardless of their relationship to the victim, in any setting not limited to home and work.
- Knowledge about HIV prevention or transmission–respondents that knew and answered at least three ways of HIV prevention or transmission correctly with no misconception were considered as knowledgeable.
- Comprehensive HIV and AIDS knowledge: defined as correct knowledge of three ways to prevent HIV and rejection of three misconceptions about HIV.

## 3. Results

### 3.1. Sociodemographic Characteristics

A total of 620 students agreed to participate in the study making a response rate of 97.8%. Mean age of participants was 18.11 (SD±1.28) years, and vast majorities (98.7%) were never married. About two-thirds (67.4%) of the participants were living with their families whereas 70.8% perceived their family's economic status as medium compared to the community they were living in (Table 2).

### 3.2. Knowledge about HIV/AIDS

Almost all respondents (99.8%) knew at least one way of HIV transmission and 99.4% mentioned unsafe sex. On the other hand, 9.8% of respondents believed that sharing household utensils or insect bite can transmit HIV. Regarding methods of HIV prevention, almost all (99.7%) respondents knew at least one and nearly two-thirds (63.7%) of them said condom prevents HIV. In general, almost half (43.9%) of the respondents had a comprehensive knowledge about HIV/AIDS (Table 3).

### 3.3. Sexual Behavior

More than half (55.3%) of the girls ever had at least one sexual partner in their lifetime (a mean of 2.36) and 75.5% of them had a boyfriend during survey. Among respondents who ever had a boyfriend, 35.3% had at least 10 years age gap with their first partner. About fifth of the study participants had sexual intercourse prior to the survey. More than one-third (35.4%) of sexually active participants had their sexual debut before the age of 18.

### 3.4. Magnitude of Transactional Sex

More than half (54.6%) of sexually active respondents were involved in TS with “sugar daddies”, whereas a fifth (18.7%) had the intention. About half (52.9%) of respondents reported to have had at least one female school friend who had experienced TS.

### 3.5. Characteristics of “Sugar Daddies” and Reason for Transactional Sex

Students who dated “sugar daddies” reported that their sexual partners were about twice as older compared to their age. Most (83.1%) of respondents' older partners were perceived wealthier. In-depth interview also revealed similar explanations as most interviewees said that “sugar daddies” were older and had full pockets. Almost all interviewees described “sugar daddies” as married.

Among respondents involved in TS, 78.9% reasoned out money and/or gift. However, all of them had received money and/or materials from “sugar daddies” in exchange for sex. Girls used the money they get from “sugar daddies” to buy clothes, shoes, jewelries, and only small proportion (9.3%) used it for school fees.

Participants in of the in-depth interview also explained the primary motivation of engaging in TS to be financial gain. Most respondents said young men of their age are usually at school, have a difficulty of making money, and mostly approach them for a free sexual favor. The money these girls charge “sugar daddies” could range from 120 Ethiopian Birr (5 USD) to 500 ETB (20 USD). One in-depth interview participant indicated the situation as follows:

*“Family thinks us only need stationeries to learn, and the money we are given goes for this purpose only not even for transportation. But we need to enjoy life the way other rich students do. You know, we want to have fun as a teenager. So, we don't care about his age, what matters most is his wallet. You may not believe where they took us……the fanciest places of the town.”* (Respondent 5, Age = 20)

Most of the interviewed girls nvolved in a sexual relationship with older men in order to get money for in essentials like chilling with friends and brand new clothes. The students have also mentioned negative peer influence as a driving force for engaging in TS.

*“Family cannot fulfill all things I want to have. When I saw my friends look nice, wearing brand clothes, I also want to have one. Plus sometimes my friends may say ‘what happened to you? Please don't kill your time rather use your body and make some money' so these things initiate me. In some occasions, my friends might ask me to eat food in a restaurant and if I do not have money, I do not have the gut to go in there. I don't want to be separated from my friends; it is so weird. So I got in transactional sex and managed to earn some money.”* (Respondent 3, Age = 18)

“Sugar daddies” might have different reasons for engaging in extramarital sex with a younger female partner. However, most of the interviewed girls shared similar perception on why older men are interested in TS with them. Their opinions go around the men's marriage, and the majority of participants used the following expression:

“As a married person, he [sugar daddy] has a lot to lose if his family knows the case. However, they do it because it is exciting and exhilarating. Maybe he is stuck in an unhappy marriage; maybe he has been married for years and had a family. He cannot divorce, because he still has some sense of love to his wife, and couldn't see her get hurted.”

### 3.6. Trends and Ways of Getting “Sugar Daddies”

“Sugar daddies” use different strategies to attract girls; mostly, they come near to school and ask girls for direction as a stranger or as someone who needs a tour guide. However, there are also girls who actively hunt for “sugar daddies”; one respondent explained her first occasion as follows:

*“I heard about a woman that match older wealthy men with attractive young girls, and I dropped my photos and personal contacts there specifically for monetary gain. A lucky girl can get showered with gifts, and enjoy meals at best tables in town. My dates began offering gifts; first, I was horrified, it seemed so immoral. But, once I'd convinced myself of moral justification, I started to enjoy it.”*(Respondent 10, Age = 22)

Some school girls were not actively approaching “sugar daddies”, instead “sugar daddies” made their way to reach these girls. Some were even victim who was unaware of the sexual conduct rather than willingly engaging in sex with an older man. One participant narrated her experience as follows:

*“There was a friend of mine whom I loved much... She never wore what she wore yesterday. She took me to her house and told me her little secret, how she earned money. One day she arranged a reason to go out and brought me a “sugar daddy”. He obliged me to drink until I got tired. Then, I do not know where he took me and what he did; I woke up naked. I was scared and cried as I did not have any sexual experience before. I called my friend, and she took care of me. After a month of dilemma, my friend convinced me to date sugar daddies and make money like her.” *(Respondent 1, Age = 16)

### 3.7. Sexual Network and Risky Behaviors in Transactional Sex

Most (78.9%) of the respondents who were engaged in TS had a concurrent sexual partner other than the older one. Majority of their parallel partners were young employees (43.7%) followed by peer male students (23.9%) and the rest 11.3% had both young employees and students simultaneously. Only 8.9% of the respondents' partners were aware of their girlfriends' sexual relation with “sugar daddies”. When respondents asked about the duration of transactional relationship with older men, most of them said it did not last long.

*“Sometimes we became familiar with one man and ignored condom. However, I personally don't like to stick to one man even if he is rich. With regards to getting money, it is good to change them. I do not like a fixed relationship, and I do not have a regular boyfriend but sometimes I went out with my classmates. But it was not a fixed one and they knew it very well; we did it because we knew each other.”* (Respondent 2, Age = 21)

The qualitative result also revealed that many young women have a concurrent relationship with older men and other fixed boyfriend among same age partners.

*“... Yeah, I have a boyfriend, a third-year university student. He is the one that I am serious with…we do sex without a condom. For instance, he recently came to me to say goodbye as he was going to see his family who lives in other city. By then, we could not get a condom and did sex without it.”* (Respondent 6, Age = 23)

### 3.8. HIV Risk Perception and Condom Use

Despite perceived advantages girls associate to it, TS carries substantial risks. Majority of respondents understood they were practicing risky sexual behavior, but they never bother about risks such relationship could bear as their current benefits outweigh the risk. Three-fourths (76%) of participants noted that they did not know their older partners' sexual history.

“*Of course, this work is so risky and would expose us to HIV. My friend was infected. However, she is still in the business and had never disclosed her status.”* (Respondent 4, Age = 17)

Only 18.5% of sexually active students had used condom consistently, whereas 28.5% had never used at all. Trusting their partners and partner refusal were the main reasons. Most (91.5%) of the girls who engaged in TS had experienced condom use, but only 19.7% were found to have had consistent use. Only 22.7% of respondents who had multiple sexual partners had used condom consistently. Reasons for not using a condom were partner's refusal and trust. Similarly, 26.7% of students who dated “sugar daddies” had never negotiated condom and the two main reasons were to get more money (55.6%) and scared of asking condom use as the men would be angry with them (33.4%). Sexual violence was also a common event in TS as reported by 77.5% of participants. The most common (74.5%) type of sexual violence was forced sex without condom.

In-depth interview participants explained that even if they had recognized the risk of TS, they were often not in a position to negotiate condom as most “sugar daddies” enjoy sex without it. Thus, it was unlikely for the girls to convince those men and use condom consistently. One informant explained the situation:

*“... Most of them did not want to use condom. Their reasons were they got tired quickly, it did not give them comfort, and some even accused me of not trusting them. Despite my effort to convince them, in case they use force; obviously, I cannot win them back; and there were times when I failed. Some guys never want to hear about condom.”* (Respondent 3, Age = 18)

### 3.9. Substance Abuse

A quarter (25%) of the respondents had used at least one type of substance; of them, 99.35% consumed alcohol, and 21.94% chewed chat. Among respondents who had sex with older men, 71.8% had consumed alcohol, and 31% chewed chat regularly. As respondents explained, sugar daddies wanted them to get drunk to forget their age gap and relax with them.

*“Drinking alcohol, chewing chat and smoking cigarettes are the common ones. There are some more like Shisha  .... We use substances to avoid fear. It gives us strength to do hardcore. It is like agreement package”* (Respondent 4, Age = 17)

### 3.10. Families' Awareness about Students' Sexual Relation with Older Men

Only 11.3% of girls' parents knew the transactional sexual relationship their daughter had with older men. Almost all interviewees explained that there is no way families could see them in action as they act humble in their neighborhood. Some tell their parents that they were at library or doing an assignment in their girlfriends' home when they have planning to meet older partners.

*“They may hear some rumors which had no proof. There was a time that my dad used to hear some gossips from neighbors. When he asked me I yelled back saying 'do not you trust me?' I convinced him so. You joke about it and it is over. You must be decent at home though; always obedient. Most of us fake being moms' daughter”* (Respondent 7, Age = 19)

### 3.11. Factors Associated with Transactional Sex

Multivariate analysis revealed that age, perceived low economic status, being orphan, and substance abuse showed a significant positive association with the practice of TS with “sugar daddies”.

Those in the age group of 20-24 years had about seven times [OR: 6.87; 95% CI: 3.48-13.58] greater odds of being involved in TS with “sugar daddies” than those in 15-19. Similarly, respondents who had lost both parents had about three times [OR: 2.99; 95% CI: 1.14-7.84] greater odds of practicing sexual relationship with older men. Participants from perceived middle income family had two times [OR: 2.54; 95% CI: 7.80-82.76] higher odds of involving in TS than those from a wealthy family. Respondents who used substances were about seven times [OR: 6.9; 95% CI: 3.7- 12.78] more likely to engage in TS. On the other hand, comprehensive knowledge about HIV/AIDS showed no significant association with involvement in TS (Table 4). Qualitative findings of the study also supported the results of the quantitative survey.

## 4. Discussion

The present study found that majority of the participants started sex at a younger age. A substantial number of girls in this study had multiple and concurrent sexual partners including with“sugar daddies” and had unsafe sex. Substance use often facilitated these encounters, which was also reported in the previous studies [28–30]. This risky sexual behavior put the girls and their entire sexual network including schoolmates at risk of HIV [31]. In accordance with previous research, most girls had TS for material gain, and peer influence was also a great motivator for these risky behaviors [32].

About a fifth of respondents (21%) had experienced sexual intercourse, and 35.4% of them had their first sex before they turned 18. This rate is almost similar with studies done in Agaro and Addis Ababa where 25% [21] and 38% [22] of school youths had sex before the age of 18. Median age at first sexual debut in this study was 17 years, and it is in agreement with the national level (16.1 years) [3] and studies from Bahirdar (16.9 years) [26] and South Ethiopia (17 years) [27].

With the increase in age of marriage, the involvement of girls in high school also has increased. In such conditions the chances of girls to have their sexual debut before marriage is high as documented elsewhere in Ethiopia [33]. Pressure to be sexually adventurous and prove manhood is quite pervasive in Africa. These norms encourage older men to have sexual relations with younger women, which allow them to have more sexual partners than women, and increase acceptance and justification of violence against women. Therefore, it is not shocking that men force sex and have multiple concurrent relationships outside wedlock [34]. About tenth (11.5%) of students were involved in sexual relations with older men. This finding is lower than the study conducted in South Africa, 25% [20] among similar grade students. This result might be underreported due to cultural influences that stigmatize girls having sex before marriage especially for financial or material benefits.

Grievance for a fancy lifestyle has motivated girls to engage in multiple concurrent partnerships with older men primarily for economic reasons [14, 35, 36]. Older men have a stronger socioeconomic position than young girls, enabling them to use money/gifts as leverage for sex. The material exchange accompanying sexual encounters may be interpreted as a loving gesture, but it may also express an unloving and calculating relationship, meaning most girls seek TS with older men purely for monetary gain. Studies in Sub-Saharan Africa show adolescents are vulnerable to socioeconomic deprivations and sexual and reproductive health risks that are often associated with poverty. Thus, students from perceived wealthy family were less likely to be involved in TS than middle- and poor-income families. Previous studies reported the association between poor economic status and risky sexual behavior [6, 13, 37] that family's income predicted girls' sexual relations with older men. Studies done in South Africa (80%) [20], Uganda (85%) [38], and Kenya (78%) [39] correspondingly found the same result that girls dated older men for financial or material benefits or both. The in-depth interview also strengthened this finding as students involved in sexual relationship with “sugar daddies” mentioned exchange of money and/or material gifts as the main reason. Qualitative studies that were done in USA [37] and Tanzania [13] similarly revealed that a girl does not engage in TS without money or other material benefit. The second reason to have sex with older men was peer influence which was also supported by the study conducted in South Africa among grade 11 and 12 students [20]. Such relationship with an older man (who is more likely to have a steady income) can provide the girls access to material wealth such as expensive clothes and shoes.

It is well documented that the number and types of sexual partners influence the risk of acquiring HIV. The practice of multiple sexual partners is among major predisposing factors for the infection [40]. Around 35% of sexually active respondents had more than one sexual partner in their lifetime which is comparable with a study from Tanzania, 37% [14]. What is worse is that still girls who reported involvement in TS counted more than five partners in their lifetime; huge at their age but expected as this type of relations does not last long. They date several men to get more financial benefit in exchange [13]. In-depth interview participants also supported this finding declaring trend of concurrent or sequential multiple sexual partners among students who dated older men for more financial or material support. Qualitative studies from Tanzania [13] and USA [37] also found the same outcome.

Unprotected sexual intercourse is one of the major risk factors that predispose individuals to HIV/AIDS [41]. In this study, only 19.7% of girls who involved in TS used condom consistently. In other words, 80.3% of students who practiced sex with older men did not use condom at all or had inconsistent use. This finding is lower than the studies done in Uganda [42] and Benin [43], where 34% of girls who dated “sugar daddies” had used condom consistently. Only 22.7% of students with a positive history of multiple sexual relationships had consistent condom use, higher than a study done in Addis Ababa 13.2% [44].

Subordinate position of women may force girls to endure abusive and violent relationships with older men in order to secure economic gains. A significant association exists between the large age gap and unsafe sex practice. Those who dated older sexual partner with ten or more year's gap had decreased odds of condom use than those who had partner less than ten years gap. This finding is in agreement with a study from Kenya that revealed the negative influence of higher age gap on condom use [7]. This was also evidenced by other studies [6, 13, 45, 46]. The qualitative findings of the present study also explored that TS with older men had lowest probability of condom use. Young girls will do whatever their older partners asked for to please them and earn more money. Even if the girl wants to use condom, power imbalance between them will leave her with no option than sacrificing herself and satisfying his interests.

Substance abuse was also related to an increased likelihood of TS, and the association is explained by various studies [47–52]. This finding may reflect the cross-sectional nature of the study and differences in a time frame for measures, as it was not possible to determine the timing of substance use in relation to TS involvement. Thus the fact that they frequently cooccur does not necessarily mean that substance use causes TS; the causal direction could be the opposite. Students could be using substances as a coping mechanism in response to experiencing TS [53]. Meaning, substance use could be associated with TS both as a risk and coping mechanism.

It is believed that knowledge about HIV/AIDS could promote safe sexual behaviors. Contrarily, this study found no significant association between HIV/AIDS knowledge and TS with older men. As participants of in-depth interview stated that they engaged in TS knowledgeably as financial and material gain outweighs to them than the risks. A study done in Kenya also revealed that young girls who had been involved in TS with older men rank other needs above the risk of HIV infection [54].

### 4.1. Limitations

This study shared the limitations of cross-sectional studies: the causal relationships between variables cannot be determined. We relied on participants' self-reports and there is a possibility of social desirability bias; hence, girls may have underreported sexual experiences [55].

## 5. Conclusion

Students engaged in TS were at risk of HIV infection due to subsequent and concurrent multiple sexual partners and inconsistent condom use. Girls engaged in such type of sexual relation were not only at risk of HIV and other STIs for themselves, but they put their partners and the youth society in general at risk while they also carry the risk of having unwanted pregnancy. While empowering girls and their families to generate adequate income is important for the long run, equipping girls with life skills that help them resist negative peer influence, negotiate safe sex, and know their HIV status should be done as a short term goal. Schools and families also need to collaborate and discuss problems among students to control the risk associated with TS and increase girls' sense of self-worth. Future longitudinal studies are also needed to examine causal associations and potential mediating influences of risk and promotive factors on TS.

## Figures and Tables

**Table 1:** Codes and categories identified in the qualitative analysis of preparatory students in Hawassa, south Ethiopia, 2011.

Categories	Codes
(1) Magnitude and way of practicing transactional sex with older men	Accessible
	Becoming common
	Get on street
	Looking for victims
	Pre-planed

(2) HIV risk perception and transactional sex with older men	No history of every one
	Insist for unsafe sex are victims
	Easily expose for HIV/risky practice

(3) Multiple sexual partners	Concurrent younger boyfriend
	Sequential sugar daddy partner
	Fixed young boyfriend

(4) Condom use and negotiation power	Don't use for young boyfriends
	Inconsistent
	Difficult to convince older men
	Bagging for condom use
	Older men dislike condom
	Deliberately break the condom
	Develop trust

(5) Substance use	Alcohol consumption
	Addict to chat
	Smoke cigarette
	Use Shisha
	Make fearless and strength for hardcore

(6) Features of “sugar daddies”	Rich/lots of money/Pay big money
	Married
	Double age difference

(7) Context and reason for sex with “sugar daddies” partners	Need to get everything
	School fees
	Secure earning/more money
	Not ask the family
	Gives a relieve
	Sympathy, generous
	Use to buy top-up

(8) Families awareness	Not see/secretly
	Out of village
	Quite descent
	Convince them
	Private room
	Obedient at home
	Trust worth

**Table 2:** Socio-demographic characteristics of female preparatory students in Hawassa, South Ethiopia, 2011 (n=620).

Variables	Frequency (%)
*Age*	
15-19	545 (87.9)
20-24	75(12.1)
*Religion*	
Orthodox	281(45.3)
Protestant	279(45.0)
Muslim	29(4.7)
Catholic	13(2.1)
Others	18(2.9)
*Student's marital status*	
Never- married	612 (98.7)
Married/Co-habitant	7 (1.1)
Divorced	1 (0.2)
*Parents marital status*	
Married and in union	488 (78.7)
Divorced	49(7.9)
Widowed	60(9.7)
Mother and father died	23 (3.7)
*Currently live with*	
Husband/cohabitate	8 (1.3)
Within families	567 (91.4)
Alone/friends	45 (7.3)
*Number of siblings*	
Less than 2 siblings	42 (6.8)
2-4 siblings	383(61.8)
Above 4 siblings	195 (31.4)
*Birth order*	
Less than 1 birth order	265 (42.7)
2-4 birth order	179(28.9)
Above 4 birth order	176 (28.4)
*Perceived family's economic status*	
Poor	81 (13.1)
Middle	439 (70.8)
Rich	100 (16.1)
*Family's residence*	
Hawassa	475 (76.6)
Outside Hawassa	145 (23.4)

**Table 3:** HIV knowledge and perception of female preparatory Students in Hawassa Town, South Ethiopia, 2011 (n=620).

Variables	Frequency (%)
*Mode of HIV transmission*	
Unsafe sex	616 (99.4)
Sharing sharp material	554 (89.4)
Contaminated blood transfusion	485 (78.2)
MTCT	476 (76.8)
Sharing home utensils	47 (7.6)
Insect bite	30 (4.8)
*Availability of treatment to cure HIV*	
Yes	233 (37.6)
No	387 (62.4)
*HIV prevention method*	
Abstinence	445 (71.8)
One-to-one relation	461 (74.4)
Consistence condom use	395 (63.7)
Avoid contaminated blood transfusion	329 (53.1)
*Perception about a health looking person can have HIV*	
Yes	582 (93.9)
No	38 (6.1)
*Previous HIV risk behavior*	
Yes	93 (15.0)
No	527 (85.0)
*Comprehensive knowledge about HIV/AIDS*	
Good	272 (43.9)
Poor	348 (56.1)

**Table 4:** Multivariable analysis on factors associated with transactional sexual relationship with older men among female preparatory students in Hawassa, South Ethiopia, 2011 (n=620).

Variables	Transactional sex		Crude OR	Adjusted OR
	Yes	No	[95% CI]	[95% CI]
	n (%)	n (%)		
*Age in years*				
15-19	42 (7.7)	503 (92.3)	1	1
20-24	29 (38.7)	46 (61.3)	2.5(1.6,3.8)	*4.82(2.41-10.35) * **∗**
*Parents' marital status*				
Married in union	54 (11.1)	434 (88.9)	1	1
Divorced	4 (8.2)	45 (91.8)	0.71 (0.25-2.06)	0.55 (0.19-1.63)
Widowed single	5 (8.3)	55 (91.7)	0.73 (0.28-1.91)	0.54 (0.20-1.46)
Lost both parents	8 (34.8)	15 (65.2)	4.29 (1.74-10.58)*∗*	*2.99(1.14-7.84) * **∗**
*Perceived economic status*				
Poor	43 (53.1)	38 (46.9)	0.6(0.3,1.0)	1.09 (0.35-3.42)
Medium	24 (5.5)	415 (94.5)	1.7(1.0,3.0)	*5.41(7.80-8.76) * **∗**
Rich	4 (4.0)	96 (96.0)	1	1
*Substance use*				
Yes	22 (64.7)	12 (35.3)	20.0(3.38-9.04)	*5.8 (2.1-15.77) * **∗**
No	49 (8.4)	537 (91.6)	*1*	*1*
*Comprehensive knowledge about HIV/AIDS*				
Good	28 (10.3)	245 (89.7)	*1*	*1*
Poor	43 (12.4)	304 (87.6)	1.24 (0.75-2.05)	0.09 (0. 53-4.23)

*∗*Indicates significant difference at p-value < .05.

## Data Availability

The data used to support the findings of this study are available from the corresponding author upon request.

## Abbreviations

AIDS: Acquired immunodeficiency syndrome
ETB: Ethiopian Birr (currency)
HIV: Human immunodeficiency virus
SSA: Sub-Saharan Africa
TS: Transactional sex
WHO: World Health Organization.

## Additional Points

Transactional sex with “sugar daddy” is a term used to describe heterosexual intercourse between younger women and twenty or more years older nonmarital partner primarily for a monetary gain. Mixed study design was employed to explore how and why preparatory students were involved in transactional sex with older men. Around a tenth of respondents had reported involvement in transactional sex with "sugar daddy"'. These students had multiple sexual partners, and majority of them had inconsistent condom use. Students in older age group, who lost both parents and had perceived less economic status had higher odds of reporting transactional sex. In conclusion, HIV prevention programs shall focus on transactional sex with "sugar daddy" to halt HIV transmission among adolescent school girls.
